# The Relationship between Moral Climate of Sports and the Moral Behavior of Young Athletes: A Multilevel Meta-analysis

**DOI:** 10.1007/s10964-018-0968-5

**Published:** 2018-12-17

**Authors:** Anouk Spruit, Maria Kavussanu, Tim Smit, Marlous IJntema

**Affiliations:** 1 Research Institute of Child Development and Education, Faculty of Social and Behavioral Sciences, University of Amsterdam, Amsterdam, The Netherlands; 2 School of Sport, Exercise, & Rehabilitation Sciences, University of Birmingham, Birmingham, UK; 3 Graduate School of Child Development and Education, Faculty of Social and Behavioral Sciences, University of Amsterdam, Amsterdam, The Netherlands

**Keywords:** Sports, Moral climate, Moral behavior, Young athletes, Multilevel meta-analysis

## Abstract

Sports are among the most important leisure activities for youth and adolescents. Both positive (i.e., prosocial) and negative (i.e., antisocial) moral behaviors occur on the playing field. To stimulate positive sports experiences, it is important to understand which factors are related to the moral behavior of young athletes; one of these is the moral climate, that is, the socio-moral environment in which sports take place. Little is known about the overall strength of the relationship between moral climate and moral behavior of young athletes, as well as the potential moderating factors of this relationship. A meta-analysis of 27 studies containing 117 effect sizes and *N* = 7726 young athletes (age < 18 years) was conducted. The results show that there is an overall significant association between these two variables (*r=* 0.40), indicating that a prosocial moral climate is related to less antisocial and more prosocial behavior, while an antisocial moral climate is associated with more antisocial and less prosocial behavior of young athletes. Two study characteristics significantly moderated this relationship: specifically, stronger associations were found in cross-sectional and in older studies. In addition, the strength of the association between moral climate and moral behavior was stronger for antisocial moral climate compared to prosocial moral climate. Finally, associations for team members were stronger than those of coaches or a broad moral club climate. Implications for further research and sports practice are discussed.

## Introduction

Sports represent one of the most popular leisure activities for youth (Ntoumanis et al. 2012). Participation in organized youth sports offers athletes many opportunities for social interactions with peers and adults, which could lead to the development of moral norms and values (Bruner et al. 2018; Rutten et al. 2011). Participants are challenged to make moral decisions about behaviors that would increase or thwart their chances of winning, and that are in line with, or contravene, the fundamental morals of sports. Because of the nature of sports activities, sports have the potential to shape the moral behavior of youth for better or worse (Rutten et al. 2008; Shields and Bredemeier 1995).

While sports are believed to promote prosocial values, such as sportsmanship and fair play, incidents of antisocial behavior on the playfield have been documented extensively (Fields et al. 2010; Hodge and Lonsdale 2011; Kavussanu et al. 2009; Rutten et al. 2011). Experiencing incidents of antisocial behavior within the sports context can have negative consequences for the sports participation of youth and limit the opportunities of effectively using sports activities as a vehicle of moral development (Al-yaaribi et al. 2016; Fraser-Thomas and Côté 2009). Therefore, it is important to understand what factors contribute to antisocial and prosocial behavior of young athletes in order to stimulate positive moral development and prevent antisocial transgressions.

One of the factors within the sports context that has been linked to moral behavior of athletes is the moral climate, that is, the socio-moral environment in which sports take place (Rutten et al. 2007). In the broader field of youth studies, the socio-moral environment created by peers, parents, and teachers in which the youth develops, is believed to be of great importance in shaping moral behavior of youth (Kohlberg 1984; Piaget 1965). In addition, findings of empirical studies highlight the importance of the moral sports climate in the moral behavior of youth (Shields et al. 2007; Rutten et al. 2011). However, to date, convincing evidence on the overall strength and potential moderators of this relationship is not available. These insights are necessary in order to be able to provide guidelines for creating a sports context that supports positive moral development of young people. Therefore, the current study aims to examine the relationship between moral climate and moral behavior of young athletes by means of a meta-analysis.

### Moral Climate and Moral Behavior

The term moral behavior refers to acts that can have positive or negative consequences for athletes’ psychological and physical well-being (Al-Yaaribi et al. 2016; Kavussanu 2012; Kavussanu and Stanger 2017). In the current study, a distinction is made between prosocial and antisocial moral behavior. Prosocial and antisocial behavior were operationalized by using the definitions utilized by Kavussanu and colleagues (e.g., Kavussanu 2008; Kavussanu and Stanger 2017; Sage et al. 2006). Prosocial behavior is behavior intended to help or benefit another, while antisocial behavior is behavior intended to harm or disadvantage another, including aggression. Examples of antisocial sports behavior are intentionally injuring an opponent, intimidating or hurting opponents, verbally abusing team members or opponents and being rebellious towards the referee (Kavussanu 2008; Sage et al. 2006). In contrast, prosocial behavior is characterized by cooperative behaviors, where people voluntarily help, take care of each other and share their resources and skills through feedback and encouragement (Kavussanu and Boardley 2009). Both prosocial and antisocial behavior can take place before, during or after the sports activities. Moreover, a recent meta-analysis (Graupensperger et al. 2018) showed that these behaviors are relatively independent, so it is important that both types of behavior are examined in sports.

The term moral climate is a broad term used to refer to a social environment in sports that has moral connotations. This includes moral atmosphere and caring climate. Moral atmosphere refers to the shared norms and values within sports context (Shields and Bredemeier 1994). This includes the shared norms and values of coaches, team members, parents, spectators, and moral club culture (Rutten et al. 2007; Shields et al. 2005). Caring climate refers to perceptions of team members on interpersonal warmth and support (Newton et al. 2007), and behaviors of “engrossment (listening, accepting, and attending), motivational displacement (honoring interests, supporting and helping achieve goals, empowering), respect (trust, sensitivity)” (Fry and Gano-Overway 2010, p. 295). The current study uses the term moral climate to refer to all of the above.

The moral climate can promote prosocial or antisocial behavior, thus it can be distinguished in prosocial and antisocial moral climate. Prosocial moral climates refer to prosocial norms, values, and behaviors within the sports context, including the presence of fair play attitudes and caring behaviors among players. Antisocial moral climate refers to a moral climate that is likely to promote antisocial behavior. Antisocial moral climates consist of shared antisocial norms and values by the social actors in the sports context, including antisocial team norms, the approval of cheating behaviors by coaches and spectators, the acceptance of injuring or intimidating opponents, and aggressive behaviors on, or around the field by team members (Guivernau and Duda 2002).

Kohlberg’s theory Kohlberg (1984) on moral development is often used to explain the relationship between moral climate and moral behavior of athletes (Jones and McNamee 2000; Shields and Bredemeier 1994; Stephens and Bredemeier 1996). Kohlberg (1984) proposed that moral reasons or beliefs are important motives to moral behavior. Believing that certain behavior is the right or acceptable thing to do, has a great motivational power to act in concordance with that belief. According to Kohlberg, individual moral beliefs in real life almost always originate in the context of group norms. Therefore, individual moral behavior can be perceived as a function of group norms (Higgins et al. 1984), explaining the association between moral climate and moral behavior in sports (Jones and McNamee 2000; Shields and Bredemeier 1994; Stephens and Bredemeier 1996).

The moral climate in the sports context is considered to be of substantial influence on moral outcomes in young athletes (Guivernau and Duda 2002; Kavussanu and Stanger 2017). The moral climate of sports teams could provide the base for moral judgments and related behavior of the team members. Numerous studies have shown that collective team, coach, parent, spectator, and club norms are related to the moral functioning and behavior of individual team members (Arthur-Banning et al. 2009; Guivernau and Duda 2002; Steinfeldt et al. 2011; Stephens et al. 1997). For example, Stephens and Bredemeier (1996) found that reported likelihood of team members to act aggressively was higher when young football players believed that other team members would play unfairly. Other research has shown that when the moral climate of the sports environment is characterized by prosocial norms, young athletes tend to show more prosocial behaviors (Rutten et al. 2007).

Several empirical studies have examined the relationship between the quality of the moral climate and moral behavior of young athletes, generally showing significant positive associations between the two variables (e.g., Shields et al. 2007; Rutten et al. 2011). The importance of a prosocial moral climate in youth sports is widely acknowledged in both research and practice. For example, on national level, organizations have been making efforts to create prosocial sports environments and ban antisocial behavior (e.g., UNICEF 2010). However, primary studies on the relationship between moral climate and moral behavior of young athletes show inconsistencies regarding the strength of this relationship, ranging from small (Bolter and Kipp 2016) to large effect sizes (Kavussanu and Spray 2006). To date, the results of primary studies on moral climate and moral behavior of young athletes have not yet been summarized quantitatively. Thus, the overall strength of the relationship between the moral climate and the behavior of young athletes is unknown. In addition, there is a lack of knowledge on the moderators of the relationships between moral climate and moral behavior of young athletes. This knowledge would allow researchers and practitioners insights on the implications for theory and practice to enhance the moral development of youth within the sports context.

### Potential Moderators

In order to fully understand the relationship between the moral climate and the moral behavior of youth, it is important to assess potential moderators of this relationship. The strength and the direction of the association between moral climate and moral behavior may depend on study, sample, sports, moral climate, and moral behavior characteristics. With regard to study characteristics, the design of the study is a potential moderator, because cross-sectional studies measure the relationship between moral climate and moral behavior at one point in time, and longitudinal studies take the developmental aspect of the association into account. Second, because it is expected that the quality of older studies is generally lower than the quality of more recent studies, as the statistical and methodological knowledge has increased largely in social research over the last decades, the year of publication could be a potential moderator. Finally, the publication status of the study (i.e., whether it was published in a peer-reviewed journal) could be a moderator, because this is an indication of whether publication bias is likely or not.

Sample characteristics, such as the proportion of males in the sample, are a potential moderator, because previous research has indicated that male athletes are more prone to aggressive behavior and that male players consider aggression and rule-violating behavior as more legitimate than female athletes (Coulomb-Cabagno and Rascle 2006; Shields et al. 2007; Tucker and Parks 2001). Consequently, it is expected that male athletes are more vulnerable to antisocial influences, because the threshold for antisocial behavior may be lower for male athletes. The proportion of athletes from Caucasian decent in the sample could also moderate the relationship between moral climate and moral behavior, because it is unknown how well the findings of previous research generalize across ethnic groups (Fredricks and Eccles 2008). Moreover, age could be a potential moderator, because research has shown that prosocial behavior and antisocial behavior increases as age increases in adolescent male football players, and similar changes occur in motivational climate (Kavussanu et al. 2006). Also, during different developmental stages, people can be more vulnerable and sensitive to the impact of social relationships and contextual influences (Kohlberg 1984; Strong et al. 2005). Fourth, the experience in the sports is a potential moderator, because the experience with sports is typically correlated with age (e.g, Kavussanu et al. 2006).

The type of sports may also influence whether the moral climate is related to moral behavior of youth (Rutten et al. 2007; Shields et al. 2007). For example, Endresen and Olweus (2005) and Vertonghen and Theeboom (2010) describe that the climate in contact sports (e.g., wrestling, power lifting, boxing, etc.) is merely built on beliefs in the value of toughness and consists of violent attitudes towards opponents, which may enhance more aggressive behavior in the sports context and everyday life. Also, in a contact-sports setting, physical aggression is rewarded with on-field success and increased prestige. Consequently, a masculine and dominant attitude could be encouraged by coaches, peers and parents in contact sports, causing a more aggressive climate and dominant behavior (Kuśnierz and Bartik 2014). Morover, stronger effect sizes for team sports are assumed, because the social influence within team sports is stronger than in individual sports.

Characteristics of the moral climate could also serve as potential moderators. First, Shields and colleagues (2007) showed that specific social actor of the moral climate (e.g., coach or team members) can influence the moral behavior of young athletes differently. Second, whether the moral climate is prosocial (e.g., caring climate) or antisocial (e.g., antisocial behavior of team members) orientated may moderate the relation between moral climate and moral behavior, because in general, antisocial influences have a larger impact on behavior then prosocial influences (Baumeister et al. 2001).

Finally, moral behavior characteristics are potential moderators. For instance, whether the moral behavior is prosocial or antisocial could be a moderator, because there are indications that the etiology of prosocial and antisocial behavior differs (Krueger et al. 2001). Prosocial behavior is believed to be largely learned by (social) reinforcement, while the development of antisocial behavior is more complex and, next to social reinforcement, involves genetic, individual, family, peer, and society influences (Bortoli et al. 2012; Carlo et al. 1999; Krueger et al. 2001). Further, whether the behavior occurred on-field or off-field could moderate the relationship between moral climate and moral behavior of young athletes. Larger effect sizes for on-field behavior are expected, because off-field behavior takes place in a different context than the sports context, and it is uncertain to what extent behavior is transferred to other contexts. In addition, research has shown differences in moral behavior between sports and other contexts such as school (e.g., Kavussanu et al. 2013; Kavussanu and Ring 2016).

## The Current Study

As indicated above, the relationship between moral climate and moral behavior in youth has not been quantitatively summarized across studies, and the potential moderators of this relationship have not been examined. This is important because such knowledge will enhance the understanding of the factors that moderate this relationship, with further implications for theory and practice. To fill in the gaps in the literature, the current study conducted a multilevel meta-analysis on the relationship between the moral climate of the sports environment and young athletes’ moral behavior. A meta-analysis can provide a summary of previous research more adequately and precisely than a narrative review (Lipsey and Wilson 2001) and is an appropriate method to quantify and analyze any inconsistencies in primary studies. A multilevel approach that allows inclusion of more than one effect size per study was used, and comprehensive moderator analyses were conducted to assess the influence of possible moderators on the relationship between moral climate and moral behavior of young athletes (Spruit et al. 2016, [Bibr CR78][Bibr CR79]; Van Den Noortgate and Onghena 2003). The multilevel meta-analytic techniques enable the use of all available effect sizes in the analyses, so all information can be preserved and maximum statistical power can be generated (Assink et al. 2015). This meta-analysis had two aims. The first aim was to examine the strength of the association between the moral climate and moral behavior of young athletes. The second aim was to test the moderating influence of study, sample, sports, moral climate, and moral behavior characteristics on this relationship.

## Methods

### Inclusion Criteria

The studies for this meta-analysis were selected using several inclusion criteria. First, articles had to examine moral climate in the sports teams and measure this by an appropriate scale, for example team norms, (socio-)moral climate, moral context, caring climate, and moral environment. The scales of moral climate had to focus on moral behavior or norms of actors in the sports context (i.e., coaches, team members, parents and spectators) or on the broad moral club culture (e.g., Rutten et al. 2007). Second, studies had to report on some type of moral behavior (e.g., prosocial behavior, antisocial behavior, aggression, etc.) of the athlete measured by a scale on past behavior (for example, questions on how often someone engaged in certain behavior in the past period of time) or intended behavior (for example, questions on how youth think they would behave in hypothetical situations). Third, only studies with a sample with a mean age below 18 were included. Finally, the included studies had to provide sufficient statistical information to calculate an effect size. Only studies reporting on bivariate associations between moral climate and moral behavior were included, because in multivariate effect sizes the set of covariates varies greatly among different studies. Therefore, combining and comparing differently adjusted effect sizes limits the ability to estimate the true overall relationship (Mulder et al. 2018).

### Selection of the Studies and Handling Publication Bias

All studies examining the relationship between moral climate in the sports context and prosocial or antisocial behavior in youth available until January 2018 were included. Five electronic databases were searched: ScienceDirect, PsychINFO (including Medline), Web of Knowledge (all databases), EBSCOhost (all databases), and Google Scholar. The search string included four combined variables: a moral climate element, a behavioral element, an age element and a sports element. For the moral climate element, the following keywords were used: “moral atmosphere”, “moral climate”, “caring climate”, “team norm*”, sportsmanship, and “fair play attitude”. For the behavioral element, the following keywords were used: behavio*r, antisocial, prosocial, aggress*, violen*, and “moral functioning”. Youth, child*, and adolescen* were used as the age element. The last keyword used is “sport*” to ensure the search was focused on studies which investigated moral climate in the sports context. Reference sections of relevant articles and publication lists of scholars, who frequently publish on this topic for qualifying studies were screened, and scholars were contacted to ask whether they would have any unpublished manuscript on this research topic.

A common problem in this type of search is that studies that do not find any significant result are often not published. Therefore, it is possible that the studies included in the meta-analysis are not an adequate representation of all previous studies that have been conducted. This creates the so-called “publication or file drawer bias” (Duval and Tweedie 2000). To prevent the problem of publication bias, unpublished studies were searched by screening all databases, including the American Doctoral Dissertations database in EBSCOhost, and by contacting authors once.

**Fig. 1:**
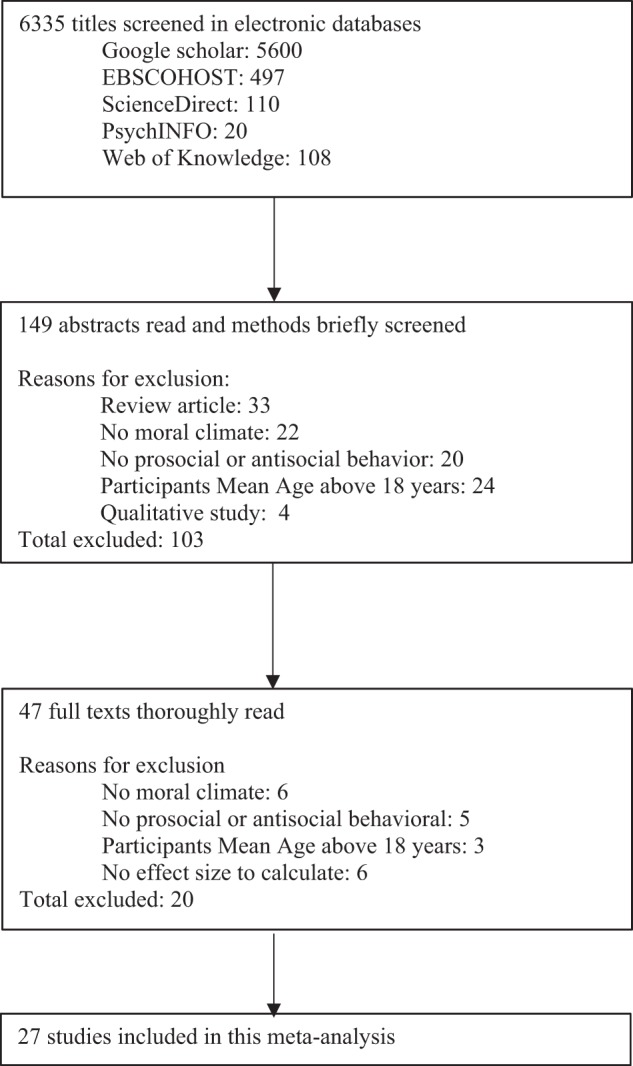
Flowchart of search results

The initial search resulted in 193 studies, selected based on the title and abstract. After deletion of double articles, the abstract and methods section of 149 studies were read. Next, 47 articles were thoroughly read to examine usability for the meta-analysis. In total, 27 studies met all the inclusion criteria and were included in this meta-analysis. Figure 1 presents a flow chart of the search of articles, while Table 1 presents the characteristics of the included studies.

**Table 1:** Characteristics of included studies

Year	N	Published	Design	%Male	% Cauc-asian	M age	Sports type	Type of moral climate	Type of moral behavior	On or off-field
Bruner et al. (2018)	100	Yes	Longitudinal	55	–	13,24	Hockey	Both	Both	On-field
Benson and Bruner (2018)	376	Yes	Cross-sectional	67	–	13,71	Ice hockey	Both	Both	On-field
Biesta et al. (2001)	260	No	Cross-sectional	59	80	14,80	Soccer & Swimming	Both	Both	Off-field
Bolter and Kipp (2016)	246	Yes	Cross-Sectional	58	87	11,80	Basketball, Volleybal, Baseball, Softball	Prosocial	Both	On-field
Bortoli et al. (2012)	388	Yes	Longitudinal	100	–	14,90	Soccer	Antisocial	Antisocial	On-field
Brand (2016)	34	No	Longitudinal	65	26	14,06	Kickboks	Antisocial	Antisocial	On-field
Chow et al. (2009)	258	Yes	Cross-sectional	39	87	–	Soccer	Prosocial	Antisocial	On-field
De Reus (2018)	43	No	Cross-sectional	100	95	16,44	Soccer	Both	Antisocial	On-field
Fry and Gano-Overway (2010)	184	Yes	Cross-sectional	21	90	13,19	Soccer	Prosocial	Prosocial	Off-field
Gano-Overway (2013)	528	Yes	Cross-sectional	46	75	12,38	Physical education	Prosocial	Both	Off-field
Gano-Overway et al. (2009)	253	Yes	Cross-sectional	50	0	11,80	Swimming, Basketball, Martial arts	Prosocial	Both	Both
Guivernau and Duda (2002)	253	Yes	Cross-sectional	70	95	15,39	Soccer	Antisocial	Antisocial	On-field
Kavussanu and Spray (2006)	325	Yes	Cross-sectional	100	–	14,58	Soccer	Antisocial	Antisocial	On-field
Malete et al. (2013)	506	Yes	Cross-sectional	77	0	15,91	Soccer	Antisocial	Antisocial	On-field
Mary et al. (2007)	429	Yes	Cross-sectional	60	–	14,70	Basketball, Volleyball, Soccer	Antisocial	Both	On-field
Miller et al. (2005)	605	Yes	Cross-sectional	60	–	–	Soccer	Antisocial	Antisocial	On-field
Orr (2012)	413	No	Cross-sectional	0	–	12,05	Softball	Antisocial	Antisocial	On-field
Romand et al. (2009)	219	Yes	Cross-sectional	100	–	14,78	Soccer	Antisocial	Antisocial	On-field
Rutten et al. (2010)	99	Yes	Longitudinal	100	46	14,62	Soccer	Both	Antisocial	On-field
Rutten et al. (2008)	331	Yes	Cross-sectional	100	51	14	Soccer	Both	Both	Both
Rutten et al. (2007)	260	Yes	Cross-sectional	59	80	14,80	Soccer & competitive swimming	Both	Prosocial	Off-field
Rutten et al. (2011)	439	Yes	Cross-sectional	100	65	15,30	Soccer, Basketball, Athletics, Taikwando	Both	Both	Both
Shields et al. (2007)	676	Yes	Cross-sectional	56	70	12,10	Basketball, Soccer, Baseball, Softball, Football, Hockey, Lacrosse	Antisocial	Antisocial	On-field
Spruit et al. (2016a, b)	155	Yes	Longitudinal	92	78	14,48	Soccer, Baseball, Basketball	Prosocial	Both	On-field
Stephens (2000)	307	Yes	Cross-sectional	67	–	12,04	Soccer	Antisocial	Antisocial	On-field
Stephens and Bredemeier (1996)	212	Yes	Cross-sectional	0	78	11,47	Soccer	Antisocial	Antisocial	On-field
Stephens and Kavanagh (2003)	267	Yes	Cross-sectional	62	–	13,10	Ice hockey	Antisocial	Antisocial	Both

### Coding the Studies

The included studies were coded according to the guidelines of Lipsey and Wilson (2001). The codebook was developed by the first, third, and last authors, and all studies were double-coded by the third and last author. In case of differences in coding, the authors discussed the coding until consensus was reached, or they consulted the first author. Potential moderators of the association between moral climate and moral behavior were grouped into study, sample, sports, moral climate, and behavior characteristics. To prevent the problem of multiple testing (Tabachnik and Fidell 2007), only moderators that had theoretical potential (as described in the Introduction), and moderators that had enough variability among the included studies were included. For example, the informant on the moral behavior outcome was not included, because there were too few studies that used informants other than self-report.

For *study* characteristics, the design of the study (cross-sectional or longitudinal), the year of publication (continuous variable), and the publication status of the study (i.e., whether it was published in a peer-reviewed journal) were coded. For the *sample* characteristic, the proportion of males in the sample, the proportion of athletes from Caucasian decent in the sample, the mean age of the participants, and the mean number of years of experience in the sports were coded. Further, different *sports* characteristics were coded as potential moderators. It was first coded if the sports were contact or non-contact sports, or that it was a mix of contact and non-contact sports. Second, a subdivision between individual, team, or mixed sports was made. Because of the limited number of non-contact and individual sports, individual sports were combined with mix, and non-contact sports with mix. With regard to the characteristics of the *moral climate*, the specific social actor of the moral climate used in the study was coded (e.g., coach, team members, or a mix/broad measure), as well as if the moral climate was prosocial (e.g., caring climate) or antisocial (e.g., antisocial behavior of team members) orientated. Finally, for the *moral behavior* characteristic, whether the moral behavior was prosocial or antisocial was coded, as well as whether the moral behavior measure consisted of ‘reported behavior’ (e.g., how often did you engage in certain behavior?) or ‘intended behavior’ (e.g., if situation X would happen, how would you behave?), and if the behavior occurred on-field or off-field.

### Calculation and Analysis

Reported statistics in the primary studies were transformed into correlation coefficient *r*, according to formulas from Lipsey and Wilson (2001); they consider *r* = 0.10 as a small effect size, *r* = 0.25 as moderate, and *r* = 0.40 as a large effect size. Effect sizes *r* were coded in the expected direction: A positive correlation indicated that a prosocial moral climate was positively related to desirable moral behavior (i.e., more prosocial and less antisocial behavior), and an antisocial moral climate was positively related to undesirable moral behavior (i.e., less prosocial and moral antisocial behavior). A negative correlation indicated that athletes engaged in antisocial behavior in a prosocial moral climate or showed prosocial behavior in an antisocial moral climate. If an article indicated that the relationship was not significant, but did not provide any statistical information, the effect size was coded as zero (Lipsey and Wilson 2001).

Continuous variables were centered on their mean, and categorical variables were re-coded into dummy variables (Lipsey and Wilson 2001). Extreme values of the effect sizes were checked (>3.29 SD from the mean: Tabachnick and Fidell 2007), but no outliers were present. Correlation coefficients *r* were re-coded into Fisher z-values for the usage of the analysis (Lipsey and Wilson 2001). For interpretation and reporting, Fisher z-values were transformed back into correlation coefficients after the analysis. The standard errors and sampling variance of the effect sizes were estimated, based on the formulas of Lipsey and Wilson (2001).

In most studies, it was possible to extract multiple effect sizes of individual studies. By using a multilevel approach, this meta-analysis accounts for the hierarchical structure of data, in which the effect sizes were nested within the studies (Van den Noortgate and Onghena 2003). This meta-analysis applied a 3-level random-effects model. The effects are accounted for three levels of variance: Level 1 contains the sampling variance for each effect size, level 2 contains the variance between effect sizes within a specific study, and level 3 contains the variance between studies (Assink and Wibbelink 2016).

The authors used R (version 3.5.0) within the foreign- and metafor-package, employing a multilevel random-effects model (Assink and Wibbelink 2016), which is often used for multilevel analyses (e.g., Assink et al. 2018; Spruit et al. 2016a, b; Ter Beek et al. 2018). To estimate the model parameters, the restricted maximum likelihood estimate (REML) was applied (Van den Noortgate and Onghena 2003). The Knapp and Hartung-method Knapp and Hartung (2003) was used to test individual regression coefficients of the models and for calculating the corresponding confidence intervals. Likelihood ratio tests were used to compare the deviance scores of the full model and the models excluding the variance parameters of level 2 or 3, making it possible to determine whether significant variance is present at the two levels (Assink and Wibbelink 2016). In case there was significant variance on these two levels, the distribution of effect sizes was considered to be heterogeneous. This indicates that the effect sizes could not be treated as estimates of a common effect size, and moderator analyses were performed. For models including moderators, an omnibus test of the fixed-model parameters was conducted, which tests the null hypothesis that the group mean effect sizes are equal. Therefore, the test statistics of the moderator analyses were based on the F-distribution.

## Results

To assess the relationship between moral sports climate and moral behavior of young athletes, a multilevel meta-analysis of 27 independent samples, 117 effect sizes, and *N* = 7726 participants was conducted. The overall association between moral climate and moral behavior of young athletes as well as the results of the moderator analyses are presented in Table 2.

**Table 2:** The overall results and moderator effects of the relationship between moral climate and moral behavior

	# study	# ES	Mean *r*	β_0_ (Fisher *Z*)	*t*_0_	β_1_	*t*_1_	*F*(d*f*_1_, d*f*_2_)
Overall association	27	117	0.40	0.43	9.78^***^			
Moderator variables								
Study characteristics								
Publication year (cont)	27	117	0.36	0.38	10.75^***^	−0.02	−4.07^**^	*F*(1, 115) = 16.58^***^
Publication status	27	117						*F*(1, 115) = 1.28
Yes	23	109	0.41	0.44	9.76^***^			
No	4	8	0.29	0.30	2.55^*^	0.14	1.13	
Type of study	27	117						*F*(1, 115) = 5.00^*^
Longitudinal	4	15	0.20	0.20	1.74^+^			
Cross-sectional	23	102	0.43	0.46	10.43^***^	0.27	2.24^*^	
Sample characteristics								
Proportion male (cont)	27	117	0.41	0.43	9.69^***^	0.04	0.32	*F*(1,1150) = 0.10
Proportion Caucasian (cont)	16	73	0.35	0.36	8.59^***^	0.20	1.46	*F*(1,71) = 2.13
Mean years of experience (cont)	15	77	0.45	0.48	7.56^***^	0.01	0.17	*F*(1,75) = 0.03
Mean age athletes (cont)	24	108	0.37	0.39	8.80^***^	0.01	0.41	*F*(1, 106) = 0.17
Sports characteristics								
Type of sports	27	117						*F*(1, 115) = 1.47
Mix	5	13	0.31	0.32	3.17^**^			
Team sports	22	104	0.42	0.45	9.56^***^	0.13	1.21	
Type of sports	27	117						*F*(1, 115) = 1.15
Contact sports	21	97	0.43	0.46	9.00^***^			
Mix	6	20	0.32	0.34	3.77^***^	−0.11	−1.07	
Moral climate characteristics								
Type of climate	23	100						*F*(1,98) = 10.22^**^
Prosocial	10	47	0.28	0.29	4.59^***^			
Antisocial	17	53	0.48	0.52	10.35^***^	0.23	3.20^**^	
Actor moral climate	27	117						*F*(2, 114) = 3.58^*^
Team member	16	50	0.49	0.53	9.29^***^			
Coach	9	37	0.38	0.40	9.07^***^	−0.13	−1.86^+^	
Mix/club culture	9	30	0.31	0.32	4.65^***^	−0.21	−2.41^*^	
Behavior characteristics								
Intended vs. reported	27	117						*F*(1, 115) = 3.24^+^
Intended behavior	10	25	0.49	0.53	6.55^***^			
Reported behavior	21	91	0.37	0.39	8.20^***^	−0.14	−1.80^+^	
Prosocial vs. antisocial	27	117						*F*(1,11) = 1.45
Prosocial	11	36	0.36	0.38	6.56^***^			
Antisocial	26	81	0.41	0.44	9.96^***^	0.06	1.21	
On-field vs. off-field	27	117						*F*(1, 115) = 0.73
On-field	23	102	0.41	0.44	9.31^***^			
Off-field	6	15	0.35	0.36	4.08^***^	−0.05	−0.62	

#study number of independent studies, #ES number of effect sizes, (cont) continuous variable, *t*_0_ difference in mean *r* from zero, *t*_1_ difference in mean *r* from reference category, *F*(d*f*1, d*f*2) omnibus test

*p < 0.05; *p* < 0.01; ***p < 0.001

### Relationship between Moral Climate and Moral Behavior

Overall, a significant, large positive association between the moral climate in the sports context and moral behavior of young athletes (*r* = 0.40; 95% CI: 0.33– 0.48; *p* < 0.001) was found. The findings show that a prosocial moral climate is related to more prosocial and less antisocial behavior, whereas an antisocial moral climate is related to more antisocial and less prosocial behavior. The likelihood ratio test comparing models with and without between-study variance (level 3) showed significant variance at the between-study level (σ²_level3_ = 0.03, χ² (1) = 24.33; *p* < 0.0001). The variance between the effect sizes within studies (level 2) was significant as well (σ²_level2_ = 0.05, χ² (1) = 945.70; *p<* 0.0001), which indicates that there is a heterogeneous effect size distribution. About 4% of the total effect size variance was accounted for the sampling variance (level 1), 57% for the variance between effect sizes within studies (level 2), and 39% for the variance between studies (level 3). Because of the heterogeneous effect size distribution, the assumption for homogeneous effect sizes was not met. Therefore, a trim and fill procedure to test for publication bias was not performed, because this procedure would not have given a reliable estimate of effect sizes (Terrin et al. 2003). However, because of the heterogeneous effect size distribution, moderator analyses were performed.

### Moderator Analyses

Several variables were examined as moderators of the relationship between the moral climate and moral behavior of young athletes. First, it was investigated whether *study* characteristics moderate the relationship between the moral climate and moral behavior of young athletes. The publication year and the type of study significantly moderated this relationship, such that larger effect sizes were found in older and cross-sectional studies. Publication status was not a significant moderator.

Next, *sample* and *sports* characteristics were examined, but none proved to be a moderator. The proportion of males, the proportion of athletes from Caucasian background, the mean age of the sample, and the mean number of years of experience with that particular sports did not moderate the strength of the association between a moral sports context and moral behavior of young athletes. Whether the sports in the studies were team sports or individual and mixed, and contact sports or non-contact or mixed, also did not moderate the relationship between moral climate and moral behavior of young athletes.

Then, it was examined whether the *moral climate* was measured as prosocial (e.g., caring climate and prosocial behaviors) or antisocial (e.g., antisocial team norms or cheating behaviors) significantly moderated the association between moral climate and moral behavior: Larger effect sizes were found for antisocial compared to prosocial moral climate. In addition, the actor of the moral climate (i.e., coach, team member, and mix/moral club culture) significantly moderated the relationship between moral climate and moral behavior of young athletes. Larger effect sizes were found for team members, compared to coach and a mix/broad moral club culture.

Finally, *moral behavior* characteristics were examined. The type of moral behavior (reported behavior vs. intended behavior and prosocial vs. antisocial behavior) and whether the behavior was on-field or off-field did not significantly influence the relationship between moral climate and moral behavior of young athletes.

## Discussion

Engaging in sports is an important leisure activity for youth and adolescents (Ntoumanis et al. 2012). Because of its social nature, sports provide many opportunities for moral decision making and moral behaviors, resulting in both prosocial and antisocial behaviors (Kavussanu et al. 2009; Rutten et al. 2011). Due to the consequences of antisocial acts on the sports experiences of youth, it is important to understand the factors that are associated with moral behavior of young athletes in order to prevent immoral behavior. Previous research has shown that the moral climate, that is, the sociomoral environment in which sports take place, plays an important role in young athletes’ moral behaviors (Guivernau and Duda 2002; Kavussanu and Stanger 2017; Rutten et al. 2008), but to date it is unknown to what extent the moral climate and moral behavior are associated. This multilevel meta-analysis of 27 independent samples, 117 effect sizes, and *N* = 7726 participants examined the relationship between moral climate in the sports context and moral behavior of young athletes. The strength of the overall relationship between moral climate and moral behavior of young athletes, and potential moderators of this association were tested.

Overall, a significant, large correlation (*r* = 0.40) was found, which indicates that a prosocial moral climate is associated with less antisocial and more prosocial behavior, while an antisocial moral climate is associated with more antisocial and less prosocial behavior of youth. These findings suggest that the sociomoral context within which young athletes’ behavior takes place can have an important influence on this behavior. In general, this relationship can be explained by Kohlberg’s theory Kohlberg (1984) on the influence of group norms on the moral behavior (Higgins et al. 1984). Moral decisions in the sports context are made in a social setting, under the influence of peers, coaches, parents, and other social agents. The current study showed that the norms and values of the social actors of the sports context are related to prosocial and antisocial behaviors of young athletes. The findings are in line with Kohlberg’s (1984) theorizing on the importance of group norms on the moral decision making and highlight the role of the socio-moral environment on moral behavior in sports.

The majority of the participants in the included samples in this meta-analysis were adolescents. The overall large positive correlation between moral climate and moral behavior in young athletes identified in this study is not directly generalizable to adult populations. Adolescence is a developmental stage that is characterized by enhanced sensitivity to social cues (Blakemore and Mills 2014; Somerville et al. 2010). Sociomoral influences in general are thus highly influential in adolescence. Moreover, although parents are often part of the moral sports climate, the moral climate in youth sports is dominated by peers and non-parental role models, such as coaches (Ntoumanis et al. 2012). Adolescence is the time in which youth begin to separate themselves from parents, as they seek emotional support from caring non-familial adults, establish relationships with peers in increased importance, and feel the need to belong and connect to groups other than the family (Fredricks and Eccles 2008). Therefore, the finding of the current study that the moral climate is strongly related to the moral behavior of young athletes and the associated theoretical explanations may be especially relevant to the adolescent population.

Various moderators of the relationship between moral climate and moral behavior of young athletes were found. First, publication year was a significant moderator. Recently published studies yielded smaller effect sizes then older studies. A possible explanation for this could be that the social environment of youth changed dramatically over the last 20 years. The social media world nowadays has major impact on the norms and behavior of youth (Elmore et al. 2017; Lin et al. 2016). With the launch of social media such as Facebook, Instagram, and Snapchat that allow the creation and exchange of user-generated content and the interaction with unfamiliar people on a large scale, unique opportunities to gather normative information from sports celebrities, online peer groups, or certain trends were added to the lives of youth (Elmore et al. 2017), above and beyond the normative influences of parents, coaches, and offline friends. Consequently, the unique contribution of the offline world to the moral behaviors of youth declined, explaining the smaller association between moral climate and moral behavior in recent studies.

The design of the study was also a significant moderator of the relationship between moral sports climate and moral behavior. Studies with a cross-sectional study design showed larger effect sizes then longitudinal studies. The moral climate in a sports team or club is not a fixed characteristic (Rutten et al. 2010), it may be influenced through different events happening at the club, on the field or in society, causing the moral climate to fluctuate. Therefore, the moral climate can be time dependent, making it more sensitive to yield significant effects for cross-sectional designs.

Third, whether the prosocial dimension (e.g., caring climate and fair play attitudes of coaches) or the antisocial dimension (e.g., aggressive behavior of the coach and antisocial team norms) of the moral climate was measured, significantly influenced the relationship between moral climate and moral behavior of young athletes. The antisocial dimension of moral climate yielded larger effect sizes than prosocial dimension. Baumeister and colleagues (2001) proposed the general law of psychological phenomena in which “bad” influences are stronger then “good” influences. Negative valanced experiences (for example, antisocial role models, negative communication, and conflict) tend to have greater impact on behavior than positive valanced experiences (for example, harmonious peer interactions, being praised, and respected; Baumeister et al. 2001). In addition, during adolescence, it is common for youth to experiment with risk taking behaviors, social boundaries, and antisocial behaviors, and it has been argued that these behaviors actually serve developmental purposes in adolescence (Blakemore and Mills 2014; Moffitt et al. 2002). Consequently, adolescents may be more sensitive to antisocial influences compared to prosocial influences, explaining why this study found that antisocial moral climate was more strongly related to moral behavior then prosocial moral climate in young athletes.

Finally, the social actor of the moral climate was a significant moderator of the relationship between moral climate and moral behavior. This study found larger effect sizes for team members, compared to coaches and a mix or broad moral club climate. This could indicate that the influence of the moral norms and behaviors of team members is larger than those of coaches and the broader club climate and underlines the important role peers play during adolescence. Indeed, peer cultures have been shown to be of great importance in shaping development and behaviors of youth, and adolescence is a time of increased sensitivity to peer influence and decreased sensitivity to adult influences (Knoll et al. 2015; Piaget 1965; van Hoorn et al. 2016). In addition, Piaget (1965) proposed that although adult authority figures could teach children about specific moral values, it is only in the context of peers that youth are truly able to put their morality into practice. This would explain the larger effect sizes for team members in the relationship between moral climate and moral behavior.

### Limitations of the Study

Several limitations of this meta-analysis should be mentioned. First, the primary studies included in this meta-analysis did not report on all potential moderators of interest. Therefore, studies with missing information on a particular moderator could not be included in that specific moderator analysis. This may pose a threat to the external validity of those findings, because not all available studies on the relationship between moral sports climate and moral behavior were included. Second, some of the hypotheses on potential moderators could not be fully tested, because there were too few or no primary studies in a particular category. For instance, too few studies reported on the relationship between moral climate and moral behavior in individual and non-contact sports. Third, in the large majority of the primary studies included in the meta-analysis both constructs were measured by the same informant and the same measurement type (i.e., questionnaires), resulting in the issue of shared method variance (Lindell and Whitney 2001). Also, it is likely that participants “project” their own behavior on others, so when they act aggressively they perceive others as acting aggressively, because they want to justify their behavior (Cho and Knowles 2013). These limitations of the primary studies may possibly cause an inflated overall effect size in the current meta-analysis.

Fifth, some categories of the moderators were filled with a limited number of studies and effect sizes. As a result, there might be a power problem in identifying moderators with a small influence. Although the difference between effect sizes of published and not published studies was small, it may be considerable (*r=* 0.12), but was not identified as a significant moderator. The same was observed for the difference between team sports and a mix of team and individual sports (*r=* 0.11). A final limitation is the fact that the authors could not check for publication bias since the assumption for a homogeneous effect size distribution was not met (Terrin et al. 2003). In case of publication bias, there could be an overrepresentation of significant effect sizes, because especially non-significant findings would be difficult to publish (Thornton and Lee 2000). However, in this study, publication bias effect is not very likely, because most studies focused on multiple predictors of moral behavior of young athletes, so non-significant results on the moral climate—moral behavior relationship are not expected to influence publication status.

### Directions for Future Research and Practice

Despite the limitations, important recommendations for future research can be made from the current study. First, because this study was not able to test all the hypotheses on potential moderators, future studies should focus on answering those questions that are currently left unanswered, for example, by comparing the association between moral climate and moral behavior among different type of sports, in different sports settings, and among different samples. Second, it is important to understand the direction of the association between moral climate and moral behavior. Currently, it is assumed that moral climate has a causal effect on moral behavior, but this has not been tested yet. Studies similar to Sage and Kavussanu (2007) may be insightful. They conducted an experiment in which athletes were randomly assigned to one of two motivational climate conditions (i.e., the extent to which the sports climate is oriented towards promoting task mastery and learning goals or social comparison and performance goals) or a control condition, and examined the effects of motivational climate in moral behavior of the athletes (Sage and Kavussanu 2007). Although the moral climate may be more difficult to manipulate, and it may not be ethically right to assign youth to an antisocial moral climate condition, it could be possible to follow youth from the start of sports activities, and investigate whether natural variation in the moral climate influences moral behavior and vice versa.

Finally, future studies should examine the predictors of moral sports climate. For example, all female teams, and good coach-athlete relationships are associated to prosocial moral atmosphere (Rutten et al. 2007). A predictor of antisocial moral climate may be teams that consist entirely of youth at risk for antisocial behavior, due to deviancy training. Deviancy training refers to “the interpersonal dynamic of mutual influence during which youth respond positively to deviant talk and behavior” (Dishion and Tipsord 2011, p. 189), and is known for its reinforcing effect on antisocial behavior in at-risk peer groups (Dishion and Tipsord 2011). This is especially important, considering the large influence of team members that was suggested in the current study. In addition, coaches with specific pedagogical skills may be able to create a prosocial moral climate (Spruit et al. 2018). Understanding the predictors of moral climate may prevent antisocial moral climate, and therefore, antisocial behaviors in the future.

This meta-analysis offers recommendations for the practice of youth sports. First and foremost, the current study emphasizes the importance of the moral climate in youth sports. More specifically, this study shows that especially an antisocial moral climate is a predictor of less prosocial and more antisocial behavior of young athletes. Therefore, youth sports organizations should make efforts to ban antisocial norms and behaviors within the sports context. Great importance has been given to the coach in preventing both on and off-field antisocial behavior (Haudenhuyse et al. 2012; Kavussanu and Spray 2006; Martin et al. 2014). Coaches should actively discourage antisocial norms and behaviors of youth (Kavussanu and Spray 2006). To facilitate coaches, youth sports clubs could consider training coaches to create an “ethical climate” by promoting prosocial behavior, to give effective feedback to youth, and are capable of acting as role models of ethical behavior for their athletes.

In addition, this meta-analysis suggested that the influence of peers is even larger than that of coaches. The behaviors and norms of peers could be influenced indirectly by means of training and education of coaches to make sure that they use appropriate educational techniques while influencing team dynamics and peer interactions (Beauchamp and Eys 2014; Coakley 2011; Conroy and Coatsworth 2006), especially considering that the majority of youth sports coaches do not have formal training in pedagogical coaching strategies or youth development (Wiersma and Sherman 2005). Moreover, youth sports organizations and policy makers could also consider influencing peer cultures directly, for example by developing programs for youth leaders or team captains to enhance positive leadership and ethical behaviors. This is underlined by research showing that adolescents have hyperresponsive neural reward systems to socially desirable peers (Somerville et al. 2010), suggesting that interventions directed at (popular) youth leaders are especially effective.

Although this study implies that team members had a relatively large influence on the moral behaviors of youth, it was also found that the moral behaviors, norms and values of coaches and the broader club culture are related to the moral behaviors of young athletes. To prevent immoral behavior of young athletes, the moral climate needs to be targeted from a broad perspective, involving all actors of the sociomoral sports environment. According to Fields and colleagues (2010), “effective interventions will likely require multifactorial approaches addressing diverse issues including peer-pressure, coaches’ influence, parental examples and expectations, media’s influence, sports figures’ influence, community and school legislation, referee enforcement of sporting rules, environmental design of sporting venues, etc.” (p. 35). In one study, small improvements of moral team atmosphere and on-field antisocial behavior were found after an intervention using forum theatre performance to address moral behavior in sports for young athletes, coaches, parents, and club managers (Rutten et al. 2010). Although research on interventions aimed at influencing moral climate and preventing antisocial behavior in youth sports is at its starting point, the current study would suggest the importance of such efforts, especially when considering the finding of larger associations between moral climate and moral behavior in cross-sectional studies. It may be that the effect of moral climate is counter-acted when youth are no longer in that environment. Changing the moral climate for the better could lead to improved moral behavior of young athletes.

## Conclusions

Despite the important role of sports in the moral development of adolescence, little is known about the factors within the sports context that are associated to moral behavior of young athletes. One of these factors that has often been linked to athlete’s moral behavior is the moral climate, that is, the sociomoral environment in which the sports take place. However, an in-depth understanding of the relationship between moral climate and moral behavior is necessary in order to stimulate prosocial and prevent antisocial behavior of young athletes. The current study is a systematic review that synthesized all available empirical studies on the relationship between moral sports climate and moral behavior of young athletes, by means of a multilevel meta-analysis of 27 independent samples and 117 effect sizes. Results suggest that the overall association between moral climate and moral behavior is large, implicating a significant role of moral norms and values of coaches, team members, and the broader club culture in the moral behaviors of young athletes. To promote positive experiences in sports activities, it is important that the sociomoral environment of the sports context is characterized by prosocial norms and values, and the absence of antisocial attitudes and behaviors. Special attention should be given to team dynamics and peer influences, as the current study showed that team members have a stronger influence on moral behavior, compared to coaches or a broad moral club climate.

## Appendix A

Figure 1
